# p21WAF1 Is Required for Interleukin-16-Induced Migration and Invasion of Vascular Smooth Muscle Cells via the p38MAPK/Sp-1/MMP-9 Pathway

**DOI:** 10.1371/journal.pone.0142153

**Published:** 2015-11-06

**Authors:** Sung Lyea Park, Byungdoo Hwang, Sun-Young Lee, Won Tae Kim, Yung Hyun Choi, Young-Chae Chang, Wun-Jae Kim, Sung-Kwon Moon

**Affiliations:** 1 Department of Food and Nutrition, Chung-Ang University, Ansung 456–756, Korea; 2 Department of Urology, Chungbuk National University, Cheongju, Chungbuk 361–763, South Korea; 3 Department of Biochemistry, College of Oriental Medicine, Dongeui University, Busan 614–052, South Korea; 4 Research Institute of Biomedical Engineering and Department of Medicine, Catholic University of Daegu School of Medicine, Daegu 705–718, Republic of Korea; University of Iowa, UNITED STATES

## Abstract

Interleukin-16 (IL-16) is a lymphocyte chemoattractant factor well known for its role in immune responses, but its role in vascular disease is unknown. Here, we explored the novel physiological function of IL-16 in vascular smooth muscle cells (VSMCs). The expression of IL-16 and its receptor CD4 was observed in VSMCs. Treatment with IL-16 enhanced the migration and invasion by VSMCs without altering the proliferative potential. IL-16 induced MMP-9 expression via the binding activity of transcription factors NF-κB, AP-1, and Sp-1 motifs in VSMCs. Among the relevant signaling pathways examined, only p38MAPK phosphorylation was significantly stimulated in IL-16-treated VSMCs. Treatment with p38MAPK inhibitor SB203580 prevented the IL-16-induced migration and invasion of VSMCs. SB203580 treatment inhibited the MMP-9 expression and activation of Sp-1 binding in IL-16-treated VSMCs, and siRNA knockdown of CD4 expression blocked the induction of migration, invasion, p38MAPK phosphorylation, MMP-9 expression, and Sp-1 binding activation stimulated by IL-16. The IL-16 induced cell-cycle-inhibitor p21WAF1 expression in VSMCs, but had no effect on the expression levels of other cell-cycle negative regulators. Finally, blockage of p21WAF1 function with specific siRNA abolished the IL-16-induced elevation of migration, invasion, p38MAPK phosphorylation, MMP-9 expression, and Sp-1 binding activation in VSMCs. Taken together, p21WAF1 was required for the induction of p38MAPK-mediated MMP-9 expression via activation of the Sp-1 binding motif, which led to migration and invasion of VSMCs interacting with IL-16/CD4. These results could provide that IL-16 is a new target in the treatment of vascular diseases such as atherosclerosis and re-stenosis.

## Introduction

Vascular disease is one of the most common causes of death in developed countries [1]. The migration and invasion by vascular smooth muscle cells (VSMCs) is a main cause of the vascular lesion formations that lead to vascular diseases including atherosclerosis and re-stenosis [1]. Matrix metalloproteinases (MMPs) play an essential role in the degradation of the extracellular matrix (ECM) components of VSMCs during plaque instability after vascular injury responses [2]. Both in vitro and in vivo experiments have demonstrated how the gelatinase MMP-9 (92 kDa) degradation enzyme of type IV collagen is a pivotal factor in the promotion of the vascular lesion process [2–5]. Many studies have reported that MMP-9 expression is stimulated by growth factors and cytokines in tumor cells and VSMCs [5–7]. In cumulative studies, the transcription factors NF-κB, Sp-1, and AP-1 are the main transcriptional cis-elements that induce the expression of MMP-9 [5, 6, 8].

Mitogen-activated protein kinase (MAPK) signaling consists of extracellular signal-regulated kinase (ERK), c-Jun N-terminal kinase (JNK), and p38MAPK [9]. Cumulative studies have shown that the MAPK signaling pathway is involved in the proliferation and migration of VSMCs [9, 10]. The MAPK signal transduction pathway is known to play a crucial role in the induction of MMP-9 expression in several cell types [11, 12]. TNF-α and interleukin-1β have been identified as inducers that control the MAPKs signaling pathway and MMP-9 expression in VSMCs [5, 12, 13].

In previous studies, p21WAF1, cyclin-dependent protein kinase inhibitor (CDKI), has played a negative role in the proliferation of VSMCs [14–16]. In addition to inhibitory modulation, p21WAF1 is known as a positive regulatory effector that is responsible for the proliferation of animal cells [17, 18]. As a result of much research effort, elucidation of the proliferative potential of VSMCs has led to an understanding of its role and significance as a cell-cycle inhibitor in the progression of vascular arterial formation [14–18]. A previous study revealed the involvement of p21WAF1 with a signaling pathway in the proliferation of VSMCs [18]. However, the regulatory mechanism of the cell-cycle inhibitors that control the migration and invasion of VSMCs remains to be fully elucidated.

Interleukin-16 (IL-16) has been identified as a lymphocyte chemoattractant factor [19]. Many studies have suggested that IL-16 transduces a signal through its receptor CD4 [20], which serves a variety of biological functions such as a growth factor of T cells and a differentiation factor of inflammatory response cells, and has been correlated with several forms of cancer diseases [21]. IL-16 induces the activation of p56^lck^, which results in the induction of PI3K/ERK/p38 cascade signaling in lympocytes [22]. Other studies have reported that IL-16 enhances translocation from the cytosol to the membrane via Protein kinase C activation in T cells [23]. In addition, IL-16 induces the activation of STAT6 through CD4 receptor in immune cell lines [24]. Only one study has used clinical serum samples to suggest that IL-16 could be one of the preventive biomarkers in coronary heart disease (CHD) [25]. However, the molecular and cellular mechanisms of IL-16 in VSMCs have not been investigated.

In the present study, we explored the roles of IL-16 and its receptor CD4 in regulating the migration and invasion by VSMCs. In addition, we introduced a novel notion whereby p21WAF1 may play a pivotal role in the regulation of migration and invasion, which is relevant to the p38MAPK signaling and Sp-1-mediated MMP-9 expression in IL-16-treated VSMCs.

## Materials and Methods

### Materials

Recombinant IL-16, polyclonal antibodies to CD-4, rat spleen lysate were purchased from R&D Systems. (Minneapolis, MN). Polyclonal antibodies to IL-16 and rat brain tissue lysate were obtained from ProSci (Poway, CA). Polyclonal antibodies to ERK, phospho-ERK, p38MAPK, phospho-p38MAPK, JNK, and phospho-JNK were purchased from Cell Signaling (Danvers, MA). SB203580 was obtained from Calbiochem (San Diego, CA). The polyclonal antibodies against MMP-2 and MMP-9 were purchased from Chemicon. Small interfering RNA (siRNA) oligonucleotides targeting CD4-1 (si-CD4-1; 5'- UAUCUUAAGAUUCUUGAUGUU-3'), CD4-2 (si-CD4-2; 5'- GAGCCAUAAUCUCAUCUGAGG-3'), p21WAF1-1 (si-p21-1; 5’-CUGUCAGUCAGUCGUAGUAUU-3’), p21WAF1-2 (si-p21-2; 5’- CCAAACGCCGGCUGAUCUUUU-3’) and scramble (5’-CUGUCAGUCAGUCGUAGUAUU-3’) were designed and synthesized from Genolution (Seoul, Korea).

### Cell Cultures

Vascular smooth muscle cells (VSMCs) were prepared from the aortas of young male Sprague Dawley rats (8 weeks old, 200-250 g), as described previously [18]. Animal experiment was performed with the approval of the Animal Care and Use Committee of Chung-Ang University. Briefly, the aortic vessels were separated under sterile conditions and were washed with Hanks' balanced salt solution (HBSS). The adventitia was removed from the aortas, which were then allowed to process in 5 ml of digestion solution (0.125 mg/ml elastase, 0.25 mg/ml soybean trypsin inhibitor, 10 mg/ml collagenase I, 2.0 mg/ml crystallized bovine albumin and 15 mM HEPES) at 37°C for 45 min. The digested cells were filtered with a sterile 100-μm nylon mesh, centrifuged at 1,000 rpm for 10 min and washed twice in Dulbecco's modified Eagle's medium (DMEM) containing 10% fetal calf serum. Then they were cultured in DMEM containing 10% fetal bovine serum (FBS). To identify the VSMCs, immunofluorescence staining was performed with a specific monoclonal antibody for smooth muscle-α-actin (Sigma Aldrich, St. Louis, MO, USA). These explants were incubated in DMEM containing 10% FBS, 2 mM glutamine, 50 μg/ml gentamycin, and 50 μl/ml amphotericin-B at 37°C in a 5% CO2 atmosphere. The cells were used for 5 to 8 passages. For use in the experiments, VSMCs were grown to 80–90% confluence and rendered quiescent by serum-starvation in DMEM without FBS for at least 24 h.

### [^3^H]Thymidine Incorporation

Proliferation of VSMCs was evaluated by the determination of [^3^H]Thymidine incorporation [18]. Quiescent VSMCs were treated with IL-16 (50 ng/ml) for 24 h. During the last 4 h of incubation, cells were labeled with 1 μCi/ml of [methyl-^3^H]thymidine (New England Nuclear; Boston, MA). Incorporated thymidine uptakes were precipitated with 10% ice-cold trichloroacetic acid, solubilized in 0.2 M NaOH, and radioactivity was established using a scintillation counter (Perkin Elmer/Wallace).

### Immunoblot

VSMCs were rinsed twice with ice-cold PBS, lysed in lysis buffer (250 μL containing, in mmol/L, HEPES [pH 7.5] 50, NaCl 150, EDTA 1, EGTA 2.5, DTT 1, β-glycerophosphate 10, NaF 1, Na_3_VO_4_ 0.1, and phenylmethylsulfonyl fluoride 0.1, 10% glycerol, 0.1% Tween-20, 10 g/mL of leupeptin, and 2 μg/mL of aprotinin), and then scraped into a 1.5 ml eppendorf tube. The lysates were obtained after centrifugation at 4°C for 20 minutes [5, 18]. The concentrations of proteins from lysates were determined by a Bradford reagent method (Bio-Rad). The cell lysates were resolved via a 0.1% SDS–10% polyacrylamide gel (SDS-PAGE) that was transferred to nitrocellulose membranes (Hybond, Amersham Corp), then blocked with 10 mmol/L Tris-HCl (pH 8.0), 150 mmol/L NaCl, and 5% (wt/vol) nonfat dry milk. The membranes were then incubated with appropriate antibodies, and the specific proteins were detected via a chemiluminescence reagent kit (Amersham Corp). The experiments were performed repeatedly at least 3 times for the immunoblot assay [5, 18].

### Conjugation of QD565 with IL-16

The carboxyl QD565 nanoparticles and *N*-ethyl-N′-dimethylaminopropyl carbodiimide (EDC) were incubated with IL-16 for 1 h at room temperature to confer the IL-16-conjugated QD565 with high efficiency coupling between the carboxyl groups and the amine [26, 27]. The ratio of the incubation reaction was 1:2:1000 for each of the QD565 particles, IL-16, and EDC. Unconjugated free IL-16 and EDC was removed by centrifugation for 15 min at 15,000 rpm, and then the QD565-IL-16 was briefly sonicated. The final forms of the conjugated particles were prepared in a Tris-borate buffer solution (10 mM Tris–HCl, 10 mM sodium borate, pH 7.4; Sigma, St. Louis, MO) [26, 27].

### Confocal microscopy of IL-16-QD565 nanoparticles in the VSMCs

Confocal microscopy was performed as described previously [26, 27]. VSMCs (8 × 10^4^ cells) were cultured with pre-coated gelatin 6-well plates, and rinsed with phosphate-buffered saline (PBS). The cells were incubated with IL-16 antibody-conjugated QD565 nanoparticles for 4 h at 37°C, and fixed with a 3.7% formaldehyde solution (Sigma, St. Louis, MO). After washing three times with PBS, the 6-well plates were mounted with medium containing 4′, 6-diamidino-2-phenylindole dihydrochloride (DAPI) solution (Vector Laboratories, Inc., CA). The fluorescence staining was analyzed via confocal laser scanning microscopy (Carl Zeiss LSM 510, Carl Zeiss, Jena, Germany).

### Wound healing migration

Quiescent VSMCs were cultured with 2 ml of growth medium in 6-well dishes (2 × 10^5^ cells/well). The confluent monolayers of the cells were damaged with a 2-mm-wide tip to create a wounded area, and were then incubated with IL-16. The cells were allowed to heal and the wounded areas were viewed by microscopy (× 40 magnification) after 24 h. The distance of cell migration to the wounded surface was estimated using Photoshop CS3.

### Invasion assay

Invasion assay was determined in ECM Matrigel-coated Transwell plates (6.5 μm, Costar, Cambridge) containing polycarbonate filters with 8-μm pores, as described previously [28]. VSMCs (2.5 × 10^4^) were added to IL-16 in 100 μL of serum-free medium and plated in the upper chamber of the transwell plate. In addition, 600 μl of medium containing 10% FBS was supplied to the lower portion. After 24 h incubation, non-invaded cells remaining on the upper surface of the transwell plates were carefully scraped off, and cells that had invaded to the lower side were fixed and stained with crystal violet, and were then measured using a light microscope.

### Zymography

Gelatin zymograhy assay was used to analyze the enzymatic activity of MMP-9 proteins [5, 28]. Quiescent VSMCs were treated with IL-16 for various time periods. Conditioned medium was resolved on 10% SDS-polyacrylamide gels containing 1 mg/ml gelatin B (Fisher). The gel was then rinsed with 2.5% Triton X-100 at room temperature for 2 h and was followed by reaction in a buffer (10 mM CaCl_2_, 150 mM NaCl, and 50 mM Tris–HCl, pH 7.5) at 37°C overnight. The gel was stained with 0.2% Coomassie blue at room temperature and incubated with destaining solution containing 10% acetic acid and 10% methanol in distilled water. The gels were photographed on a light box. Gelatinolytic activity was visualized as a white area in a dark blue field [5, 28].

### Nuclear extracts and EMSA

Nuclear proteins were extracted as described previously [28]. Briefly, VSMCs were washed, scraped, and suspended in a buffer (10 mM Hepes (pH 7.9), 10 mM KCl, 0.1 mM EDTA, 0.1 mM EGTA, 1 mM DTT, and 0.5 mM PMSF). The cells were lysed in the presence of 0.5% Nonidet NP-40. The homogenates were then centrifuged, and the nuclear pellets were extracted with an ice-cold high salt buffer (20 mM Hepes pH 7.9, 0.4 M NaCl, 1 mM EDTA, 1 mM EGTA, 1 mM DTT, and 1 mM PMSF). After centrifugation, the supernatants containing the nuclear extracts were prepared. The concentrations of proteins were measured using a Bradford reagent method (Bio-Rad).

Transcriptional activation was analyzed by EMSA, as previously described [28]. In brief, the oligonucleotides spanning the -79 MMP-9 cis element of interest were end-labeled with ^32^P-ATP by T4 polynucleotide kinase (Promega, Madison, WI). The nuclear extracts (10–20 μg) prepared from VSMCs were incubated with a radiolabeled oligonucleotide probe (10,000 cpm) at 4°C for 20 min in a binding buffer solution (25 mM HEPES buffer pH 7.9, 0.5 mM EDTA, 0.5 mM DTT, 0.05 M NaCl, and 2.5% glycerol, and 2 μg of poly dI/dC). The DNA-protein complex was resolved at 4°C on a 6% polyacrylamide gel containing a TBE running buffer (89 mM Tris, 89 mM boric acid and 1 mM EDTA). The gel was rinsed, dried, and then exposed to X-ray film for 10 h [28]. The sequences for the oligonucleotides were as follows: AP-1, CTGACCCCTGAGTCAGCACTT; NF-κB, CAGTGGAATTCCCCAGCC; and, Sp-1, GCCCATTCCTTCCGCCCCCAGATGAAGCAG.

### Transfection

VSMCs were transfected with either siRNA or scrambled siRNA using lipofectamine 2000 transfection reagent (Invitrogen) for 24 h following the protocols supplied by the manufacturer. Cells were incubated with serum-starved medium for 24 h. After one day, the cells were treated with IL-16, then studied via immunoblot, zymography, electrophoretic mobility shift assay (EMSA), invasion, and wound-healing migration.

### Cell-cycle analysis via fluorescence-activated cell sorter (FACS)

Quiescent VSMCs were cultured with various concentrations of IL-16 for 24 h. VSMCs were trypsinized, collected, and fixed in 5 ml of ice-cold 70% ethanol. After centrifugation, the cells were incubated for 30 min with PI solution (50 μg/ml propidium iodide, 50 μg/ml RNAse A in PBS). Cell-cycle distribution was analyzed via a Becton Dickinson Facstar flow cytometer supplied with Becton Dickinson Cell FIT software.

### Statistical analysis

Where appropriate, the data were recorded as the means ± SE. Data were verified using analysis of factorial ANOVA and Fisher’s least significant difference testing. Statistical significance was determined at P < 0.05.

## Results

### Detection of IL-16 and CD4 in vascular smooth muscle cells (VSMCs)

**Fig 1:**
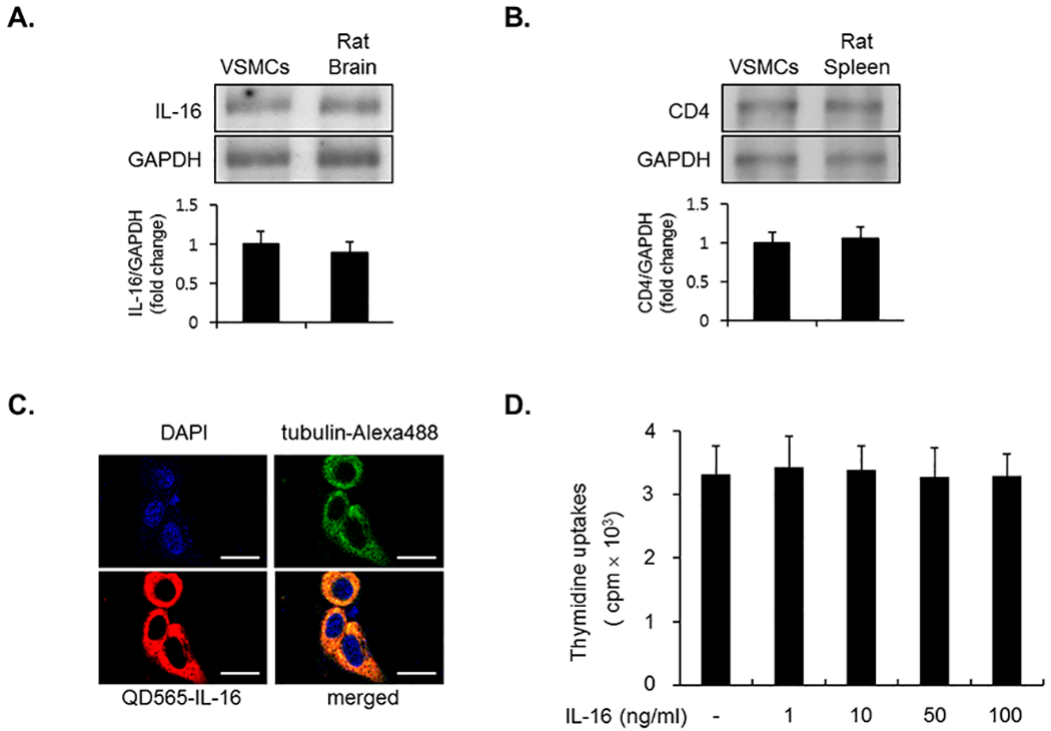
Expression of IL-16 and its receptor CD4 in VSMCs. (A, B) IL-16 and CD4 are expressed in VSMCs. Cells were cultured with DMEM medium containing 10% FBS for 24 h. Then, protein levels of IL-16 and CD4 were examined by immunoblot analysis. Lysates from rat brain tissue and rat spleen used as positive control. GAPDH expression served as the loading control. (C) Confocal staining of IL-16 in VSMCs. Cells were stained with antibody against QD565-conjugated IL-16 (red). Both DAPI and tubulin-Alexa488 were used to counterstain the nuclei (blue) and cytoplasm (green). Scale bars represent 25 μm. (D) IL-16 did not affect the proliferation of VSMCs. Subconfluent cells were cultured with serum-free medium for 24 h, prior to stimulation with IL-16 for indicated concentrations. The level of thymidine uptake was evaluated using a liquid scintillation counter. Results are represented as the means ± SE from three triplicate experiments. *P < 0.01 compared with control.

To investigate the expression of IL-16 and its receptor CD4, immunoblot assay was employed using lysates from VSMCs. The expression level of IL-16 appeared to be endogenous in VSMCs cultured with medium containing 10% FBS (Fig 1A). In addition, under the same culture conditions, CD4 was also expressed in VSMCs (Fig 1B). The expression pattern of IL-16 was confirmed in the sub-cellular regions via immunofluorescence confocal microscopy analyses (Fig 1C). In the present study, IL-16 protein was predominantly expressed in both the cytoplasm and peri-nuclear areas in VSMCs (Fig 1C). We next investigated the proliferative ability of recombinant IL-16 in VSMCs using a thymidine uptake experiment. However, the level of [^3^H] thymidine incorporation was not changed in the IL-16-treated VSMCs compared with non-treated cells (Fig 1D). These results indicated that IL-16 treatment had no effect on the proliferative effect in VSMCs.

### IL-16 treatment induced migration and invasion by VSMCs via MMP-9 expression

**Fig 2:**
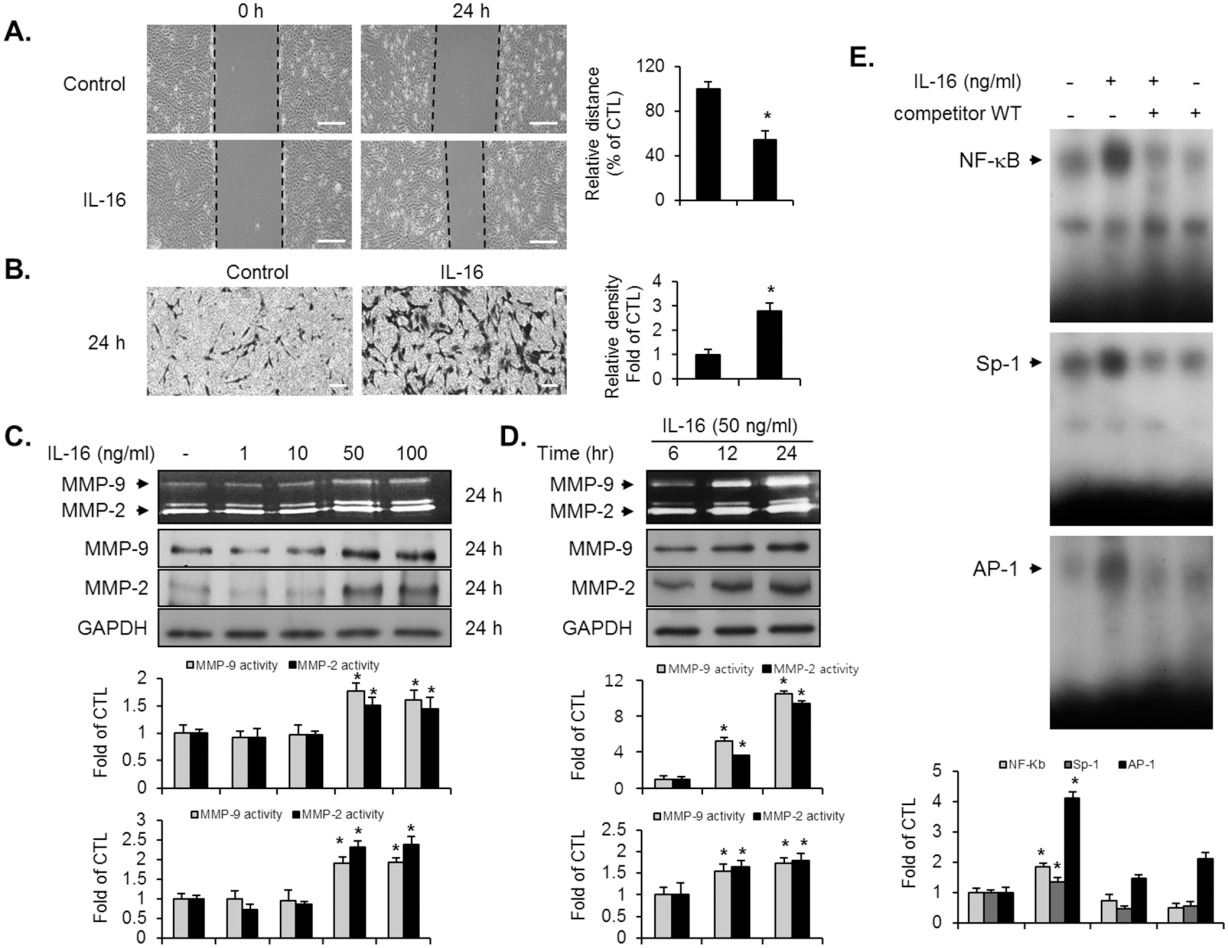
IL-16 stimulated migration and invasion via MMP-9 expression by inducing the binding activity of NF-κB, AP-1, and Sp-1 motifs in VSMCs. (A, B) The confluent cells were cultured with serum-free medium for 24 h, followed by treatment with IL-16 (50 ng/ml) for the indicated times. Wound-healing migration assay (A) was examined by evaluating the width of injury lines. The ability of invasive cells (B) was measured using a Matrigel coated chamber assay. Scale bars represent 400 μm (wound-healing) and 100 μm (invasion). (C, D) Quiescent VSMCs were treated with various concentrations of IL-16 at the indicated times. Supernatant from cells was subjected to zymography analysis for MMP-2 and MMP-9 activity. Cell lysates were determined by immunoblot analysis using antibodies against MMP-2 and MMP-9. (E) After stimulation with IL-16 for 24 h, an EMSA was performed to detect the binding activity of NF-κB, AP-1, and Sp-1 motifs from nuclear extracts using radiolabeled oligonucleotide probes. Results are reported as the means ± SE from three triplicate experiments. *P < 0.01 compared with control.

The migration and invasion by VSMCs is strongly connected to vascular lesion instability [1]. Therefore, to investigate the migratory and invasive influence of IL-16 in VSMCs, we next performed wound-healing and invasion assays. By comparison with control cells, IL-16 treatment increased the migration of VSMCs to the wounded surface at 24 h (Fig 2A). In addition, the invasion of VSMCs through matrigel-plated Boyden chambers was significantly promoted in the presence of IL-16 for 24 h, compared with that of the control cells (Fig 2B). Since MMP-9 expression is a key regulator in the migration and invasion by VSMCs [2–5], the expression level of MMP-9 was evaluated using a gelatin zymography and immunoblot assay in IL-16-treated VSMCs. After 24 h exposure of VSMCs to IL-16, increased levels of MMP-9 expression were observed in a concentration- and time-dependent pattern by comparison with control cells (Fig 2C and 2D). The protein level of MMP-9 was confirmed via immunoblot analysis (Fig 2C and 2D). In addition, the expression level of MMP-2 also increased in VSMCs that were cultured with medium containing IL-16, by comparison with control cells (Fig 2C and 2D). These results indicate that IL-16 treatment stimulates migration and invasion through the expression of MMP-9 in VSMCs.

### IL-16 promoted MMP-9 expression via transcriptional activation of NF-κB, Sp-1, and AP-1

Because MMP-9 expression is regulated by three types of transcription factors, NF-κB, Sp-1, and AP-1 [5, 6, 8], we next investigated whether IL-16 could stimulate the transcriptional activation of these factors in VSMCs. VSMCs were treated with various concentrations of IL-16 and an EMSA experiment was performed using nuclear extracts. Treatment of cells with IL-16 strongly enhanced the DNA binding activity of those transcription factors in VSMCs (Fig 2E). These binding activities were completely abolished by the insertion of excess unlabeled NF-κB, Sp-1, and AP-1 binding motifs (Fig 2E). These results demonstrated that transcriptional activation of NF-κB, Sp-1, and AP-1 binding is involved in the MMP-9 expression in IL-16-treated VSMCs.

### IL-16 stimulated the phosphorylation of p38MAPK in VSMCs

**Fig 3:**
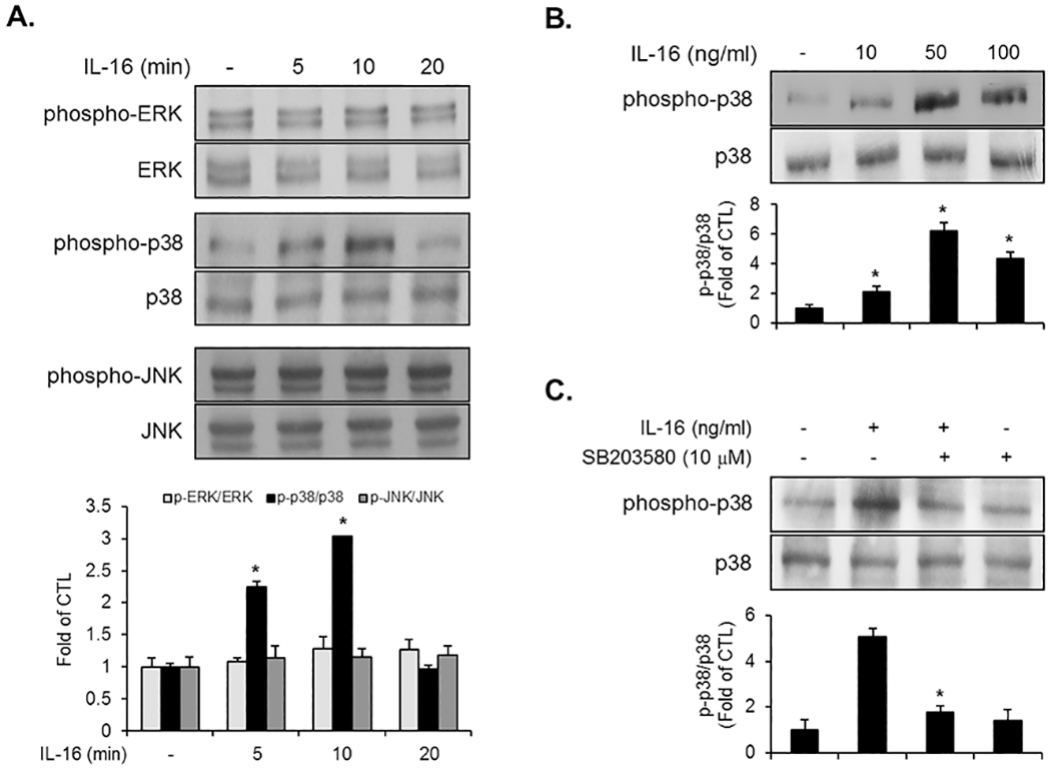
IL-16 induced the phosphorylation of p38MAPK in VSMCs. (A) Quiescent VSMCs were incubated with IL-16 (50/ng/ml) for the indicated time periods. Cell lysates were assessed by immunoblot analysis using specific antibodies against phopho-ERK1/2, ERK1/2, phospho-p38, p38, phospho-JNK, and JNK. Results are reported as the means ± SE from three triplicate experiments. *P < 0.01 compared with control. (B) Quiescent VSMCs were cultured with IL-16 for 10 min at the indicated concentrations. Cell lysates were subjected to immunoblot analysis for the phosphorylation of p38MAPK. *P < 0.01 compared with control (C) Quiescent VSMCs were pre-incubated with SB203580 (10 μM) for 40 min prior to IL-16 treatment (50 ng/ml) for 10 min. The phosphorylation levels of p38MAPK were evaluated by immunoblot. *P < 0.01 compared with IL-16 treatment.

In order to determine the intracellular signaling pathway of molecular events induced by IL-16 in VSMCs, we examined the phosphorylation of MAPKs, which are upstream signaling pathway molecules in mammalian cells. VSMCs were treated with IL-16 for various time periods. Treatment of VSMCs with IL-16 resulted in the phosphorylation of p38MAPK at 5 min (Fig 3A). The phosphorylation of p38MAPK reached a peak at 10 min in IL-16-treated VSMCs (Fig 3A). In addition, treatment with IL-16 at a 50 ng/ml concentration for 10 min induced the phosphorylation of p38MAPK in VSMCs (Fig 3B). Furthermore, the IL-16-induced phosphorylation level of p38MAPK was suppressed by the addition of specific p38MAPK signaling inhibitor SB203580 in VSMCs (Fig 3C). However, IL-16 treatment had no effect on the phosphorylation of either ERK1/2 or JNK in VSMCs (Fig 3A). These data indicate that p38MAPK signaling might be connected with IL-16-induced molecular responses of VSMCs.

### p38MAPK signaling is involved in the IL-16-induced migration and invasion of VSMCs via Sp-1-mediated MMP-9 expression

**Fig 4:**
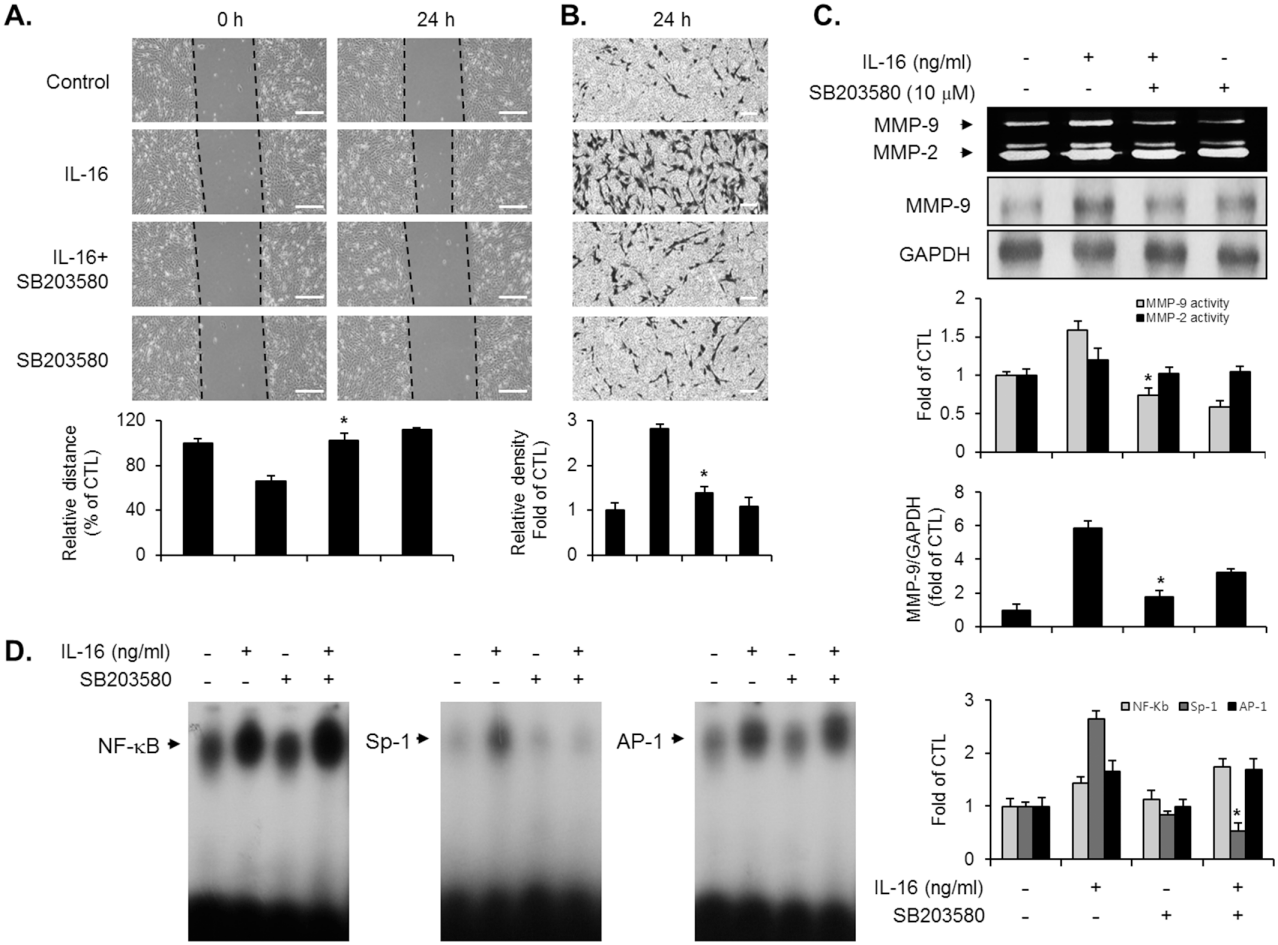
Inhibition of p38MAPK interfered with the IL-16-stimulated induction of migration, invasion, MMP-9 expression, and the Sp-1 motif activation in VSMCs. Quiescent VSMCs were pre-cultured with SB203580 (10 μM) for 40 min, prior to IL-16 (50 ng/ml) stimulation for 24 h. (A, B) Indicated cells were examined to determine the migratory capacity and invasive potential using wound-healing migration and matrigel invasion assay. Scale bars represent 400 μm (wound-healing) and 100 μm (invasion). *P < 0.01 compared with IL-16 treatment. (C) Production of MMP-9 was analyzed by gelatin zymography using the conditional medium from indicated cells. Cell lysates were examined to determine the protein level of MMP-9 via immunoblot experiment. *P < 0.01 compared with IL-16 treatment. (D) EMSA was performed with nuclear extract from indicated cells to determine the activation of NF-κB, AP-1, and Sp-1 binding motifs using radiolabeled oligonucleotide probes. Results are reported as the means ± SE from three triplicate experiments. *P < 0.01 compared with IL-16 treatment.

In order to investigate the potential involvement of the p38MAPK signaling pathway in the IL-16-induced VSMC responses, we employed SB203580 (p38MAPK specific signaling inhibitor). VSMCs were pretreated with SB203580, which was followed by IL-16. As shown in Fig 4A and 4B, pretreatment of VCMCs with SB203580 completely suppressed the migration and invasion of VSMCs induced by IL-16 to baseline levels. In addition, the inhibition of p38MAPK signaling by SB203580 decreased the MMP-9 expression in IL-16-treated VSMCs (Fig 4C). To examine the exact role of p38MAPK in the regulatory mechanism of MMP-9 expression, EMSA assay was performed in IL-16-treated VSMCs using the DNA binding motifs of NF-κB, Sp-1, and AP-1 from nuclear extract. The IL-16-induced activity of the Sp-1 binding motif was significantly abolished in the presence of SB203580 in VSMCs (Fig 4D). However, the binding activities of the NF-κB and AP-1 motifs were not affected by the addition of SB203580 in IL-16-treated VSMCs (Fig 4D). These results demonstrate that p38MAPK signaling is associated with the IL-16-induced migration and invasion of VSMCs through Sp-1-mediated MMP-9 expression.

### Role of CD4 in migration, invasion, MMP-9 expression, p38MAPK phosphorylation, and activation of the Sp-1 binding motif in IL-16-treated VSMCs

**Fig 5:**
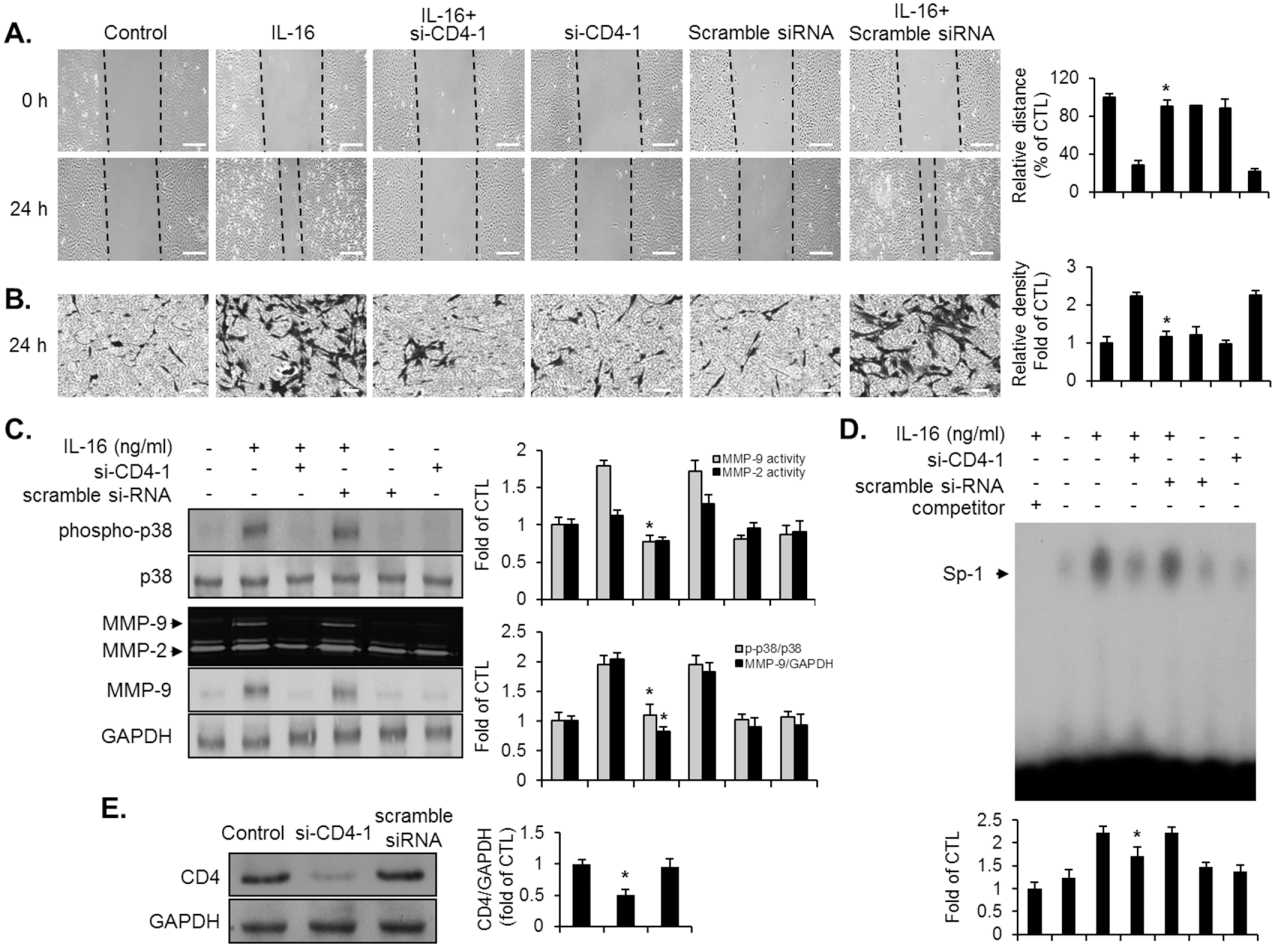
Effect of si-CD4-1 on IL-16-induced promotion of migration, invasion, p38MAPK phosphorylation, MMP-9 expression, and Sp-1 binding activity in VSMCs. Cells were transfected with either si-CD4-1 or scramble siRNA, then followed by stimulation of IL-16 for 24 h. (A, B) Indicated cells were subjected to wound-healing assay and invasion assay. Scale bars represent 400 μm (wound-healing) and 100 μm (invasion). *P < 0.01 compared with IL-16 treatment. (C) Zymography and immunoblot was used to determine the MMP-9 expression using cell supernatants and cell lysates. For the phsosphorylation of p38MAPK, transfected cells were incubated for 10 min, and then the cell lysates were analyzed via immunoblot. *P < 0.01 compared with IL-16 treatment. (D) After transfection of either si-CD4-1 or scramble siRNA, indicated cells were cultured with IL-16 (50 ng/ml) for 24 h. Binding activity of the Sp-1 motif was examined by EMSA from nuclear extracts using radiolabeled oligonucleotide probes. Unlabeled Sp-1 (1 picomole) was used as a competitor control. *P < 0.01 compared with IL-16 treatment. (E) The efficacy of silencing gene of CD4 in VSMCs. Cells were transfected with either si-CD4-1 or scramble siRNA. The protein level of CD4 was evaluated by immunoblot. GADPH was used as an internal control. Results are reported as the means ± SE from three triplicate experiments. *P < 0.01 compared with control.

CD4 receptor is required for the biological responses of IL-16 in immune cell lines [20, 21]. Because CD4 expression was detected in VSMCs, we next investigated the potential role of CD4 in the IL-16-induced responses of VSMCs. To determine whether CD4 is essential for the VSMCs to respond to IL-16, we generated a receptor-specific siRNA knockdown system (si-CD4-1), which suppressed the expression of CD4 at protein levels (Fig 5E). The knockdown of CD4 blocked the promotion of migration and invasion in IL-16-treated VSMCs (Fig 5A and 5B). In addition, the transfection of si-CD4-1 inhibited the increase in the phosphorylation of p38MAPK and the level of MMP-9 expression induced by IL-16 in VSMCs (Fig 5C). Furthermore, the enhanced IL-16-induced binding activity of Sp-1 was effectively attenuated in the VSMCs transfected with si-CD4-1 (Fig 5D). Similar results were observed in another CD4 siRNA gene (si-CD4-2) transfected cells (S1A–S1E Fig). These findings demonstrate that IL-16 promotes migration, invasion, p38MAPK phosphorylation, and Sp-1-mediated MMP-9 expression via the binding of CD4 in VSMCs.

### Cell-cycle inhibitor p21WAF1 expression is up-regulated in IL-16-treated VSMCs

**Fig 6:**
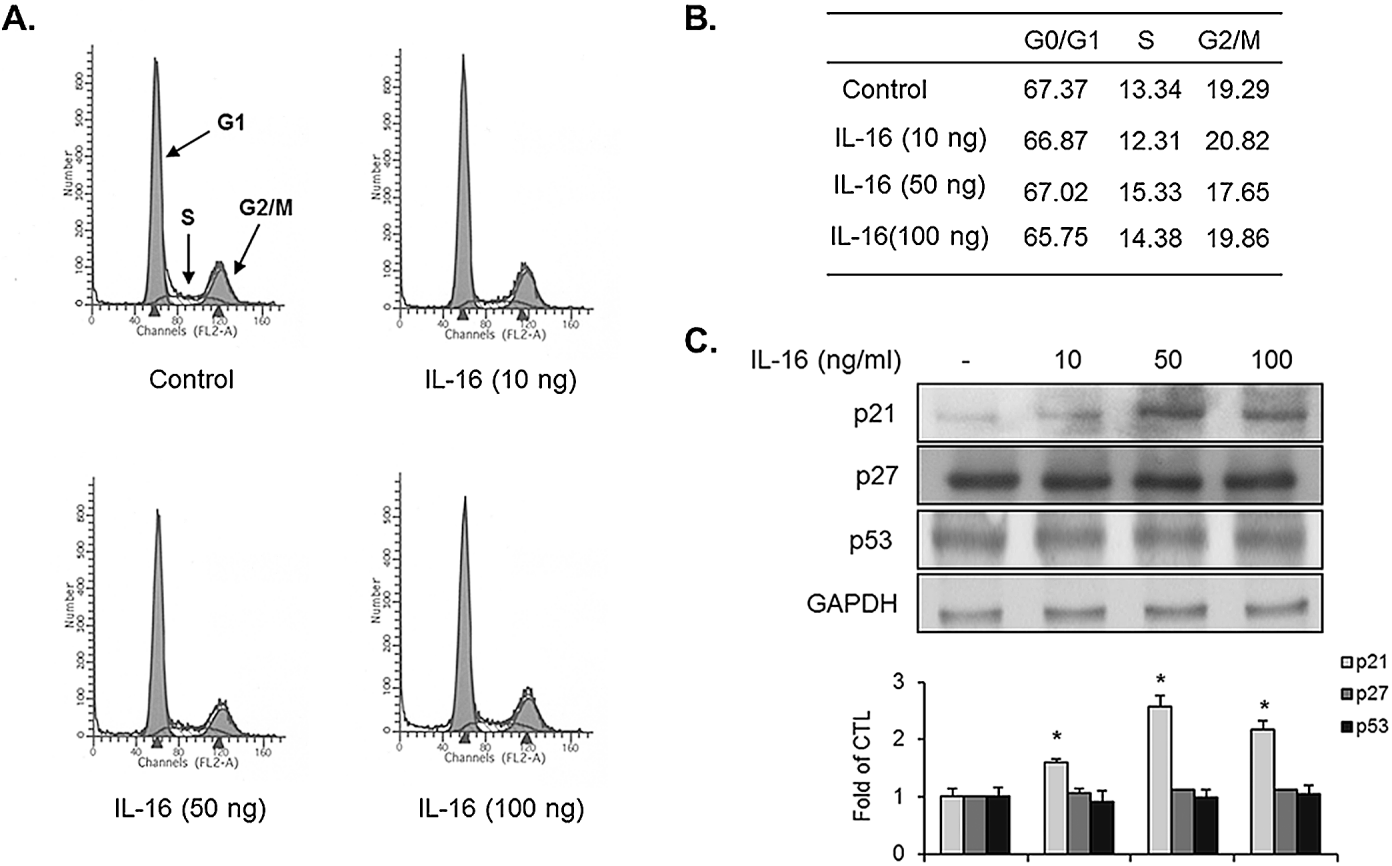
IL-16 induced expression of p21WAF1 in VSMCs. Quiescent VSMCs were incubated with indicated concentrations of IL-16 for 24 h. (A) Indicated cells were then subjected to flow cytometric analysis for the determination of cell-cycle distribution in the presence of IL-16. (B) The percentage of the cell population in each phase is shown in the table. (C) Cell lysates were obtained from the indicated cells, and then were subjected to immunblot using specific antibodies against p21WAF1, p27KIP1, and p53. The GAPDH was loaded as an internal control. Results are reported as the means ± SE from three triplicate experiments. *P < 0.01 compared with control.

To test whether IL-16 affects the cell-cycle regulation in VSMCs, we first investigated the cell-cycle population using FACS analysis. The results from the FACS approach showed that the cell-cycle distribution in the G1-, S-, and G2/M-phases was not altered by the addition of VSMCs with IL-16 for 24 h (Fig 6A and 6B). We next examined the expression level of cell-cycle inhibitors in IL-16-treated VSMCs because cell-cycle inhibitors have been deeply associated with mitogen-mediated vascular cellular responses [14–18]. To this end, VSMCs were incubated with IL-16 for 24 h. Then, we measured the expression levels of cell-cycle inhibitory regulators including p21WAF1, p27KIP1, and p53 stimulated by IL-16 in VSMCs. As shown in Fig 6C, the expression level of p21WAF1 was significantly increased in IL-16-treated VSMCs (Fig 6C). At the tested concentration of IL-16 (50 ng/ml), p21WAF1 expression was peaked in VSMCs (Fig 6C). In addition, we observed that IL-16 treatment had no effect on the expression levels of either p27KIP1 or p53 (Fig 6C). These data suggest the notion that p21WAF1 might regulate the physiological function of IL-16-mediated VSMC responses.

### p21WAF1 is an important regulator in the IL-16-induced migration, invasion, p38MAPK phosphorylation, and Sp-1-mediated MMP-9 expression of VSMCs

**Fig 7:**
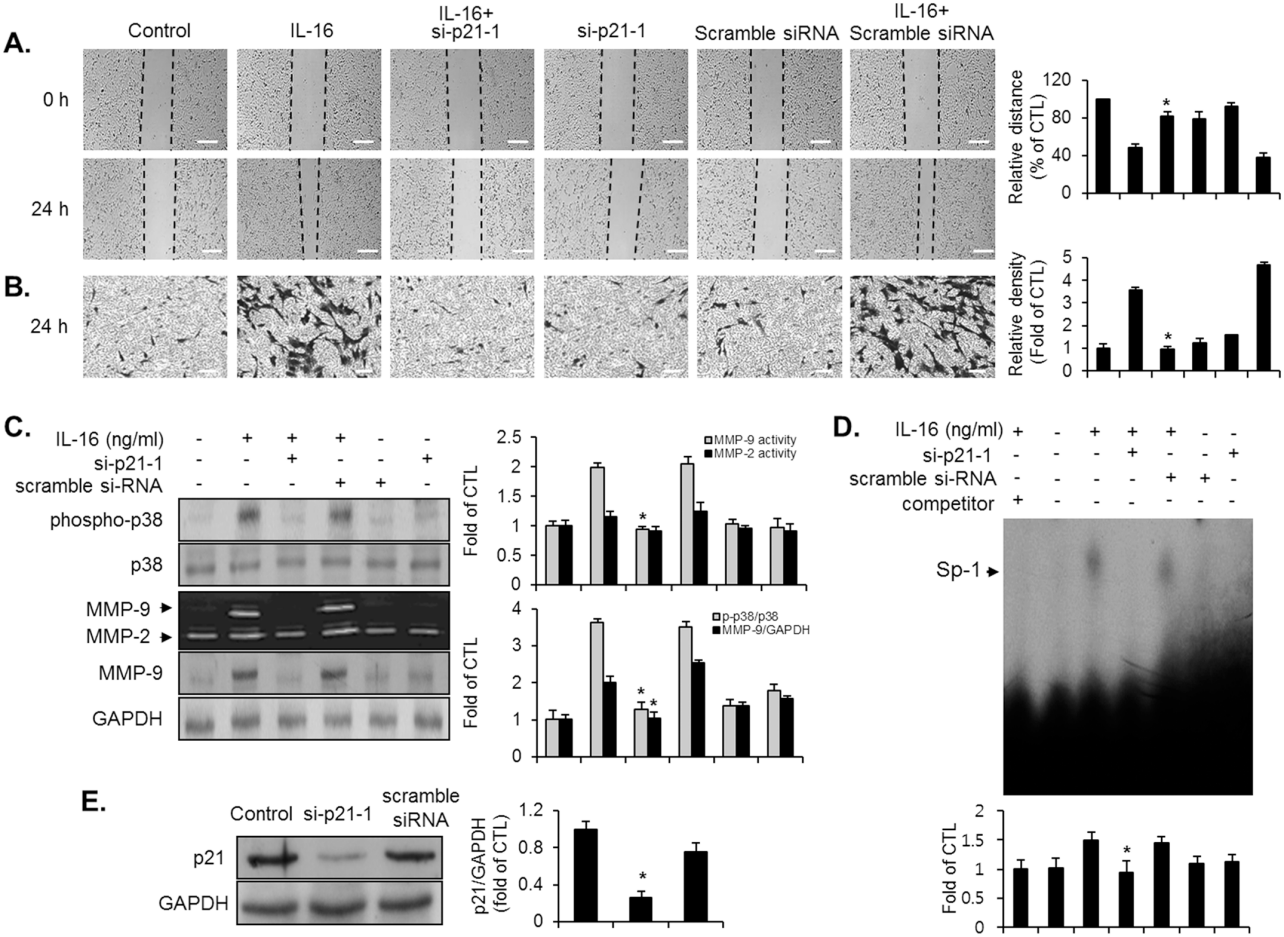
Involvement of p21WAF1 in the induction of migration, invasion, p38MAPK phosphorylation, MMP-9 expression, and Sp-1 binding activity in IL-16-stimulated VSMCs. VSMCs were transfected with either si-p21-1 or scramble siRNA, and stimulated with IL-16 (50 ng/ml) for 24 h. (A, B) Indicated cells were examined via wound-healing migration and invasion assay. Scale bars represent 400 μm (wound-healing) and 100 μm (invasion). *P < 0.01 compared with IL-16 treatment. (C) Expression level of MMP-9 was determined by zymography and immunoblot analysis using culture medium and cell lysates. For the phosphorylation level of p38MAPK, indicated transfected cells were cultured with IL-16 for 10 min. Immunoblot was performed with antibody specific for p38MAPK using cell lysates. *P < 0.01 compared with IL-16 treatment. (D) Transfected cells were incubated with IL-16 (50 ng/ml) for 24 h. Nuclear extracts were acquired from indicated cells, then subjected to EMSA for an evaluation of the binding activity of the Sp-1 motif using radiolabeled oligonucleotide probes. One picomole of unlabeled Sp-1 was loaded as a negative control (competitor). *P < 0.01 compared with IL-16 treatment. (E) Effect of knockdown of p21WAF1 gene in VSMCs. VSMCs were transfected with either si-p21-1 or scramble siRNA. The efficacy of silencing the p21WAF1 gene was assessed by immunoblot. GAPDH was employed as a loading control. Results are reported as the means ± SE from three triplicate experiments. *P < 0.01 compared with control.

We finally investigated whether p21WAF1 regulates the IL-16-mediated VSMC responses by introducing a knockdown of the p21WAF1 expression using the siRNA system (Fig 7E). Transfection of VSMCs with p21WAF1-specific siRNA (si-p21-1) blocked the increased migration and invasion in response to IL-16 (Fig 7A and 7B). Knockdown of p21WAF1 also significantly diminished the induction of p38MAPK phosphorylation in IL-16-treated VSMCs (Fig 7C). Moreover, IL-16-induced MMP-9 expression in VSMCs was abolished by the transfection of si-p21-1 (Fig 7C). To further investigate the detailed regulatory mechanism of MMP-9, as induced by IL-16, we used EMSA to focus on the transcriptional regulation. As shown in Fig 7D, VSMCs transfected with si-p21-1 reversed the enhanced binding activation of Sp-1 that had been induced by IL-16. Finally, similar results were found in VSMCs transfected with another design of p21WAF1 siRNA (si-p21-2) (S2A–S2E Fig). Based on these results, p21WAF1 might contribute to migration and invasion through p38MAPK-associated MMP-9 expression by enhancing the activation of the Sp-1 binding motif in IL-16-treated VSMCs.

## Discussion

The current study is the first to document the roles and significance of the regulatory mechanism of cytokine IL-16 in VSMCs. The main finding of the present study establishes IL-16 as a key regulator of migration and invasion by VSMCs via binding with CD4, which results in the induction of p38MAPK phosphorylation, MMP-9 expression, and Sp-1 binding activation. In addition, we also identified p21WAF1 as a pivotal molecule in modulating the physiological function of IL-16-induced VSMC responses.

The identification of IL-16 as an immune responsive factor of lymphocytes and hematopoietic cells also addressed the question of its pathological functions in solid tumors [21]. Clinical evidence from previous studies have reported mechanistic links of IL-16 to the progression of several forms of cancer such as cutaneous T cell lymphoma, multiple myeloma, breast, lung, and prostate [21]. Only one previous report has noted up-regulated levels of IL-16 in the serum samples of clinical patients bearing coronary heart disease (CHD) [25]. However, the functional role of IL-16 in vascular diseases has not previously been investigated. The present data from immunoblot and immunostaining experiments found the expression of both IL-16 and CD4 in VSMCs. Based on this finding, we assumed that IL-16 might play a critical role in the development of vascular plaque lesion formation, which was attributed to vascular diseases such as atherosclerosis and re-stenosis. Then, we launched an immediate study into how IL-16 might contribute to the physiological function of VSMCs in arterial lesion formation.

Previous studies have demonstrated that the increased expression of MMP-2 and MMP-9 is strongly associated with a formation of vascular lesions [2]. Cumulative data have shown that induction of MMP-9 expression is observed in a rat arterial injury model [2, 29]. Pathology studies from a knock-out study have suggested that MMP-9 is closely correlated with the development of vascular arterial lesion through its ability to control migration of VSMCs [3, 4]. Therefore, the role of MMP-9 regulation has been focused on the progression of vascular lesion formation. Cumulative reports have suggested that growth factors and cytokines enhance MMP-9 expression via an increased binding activation of transcription factors NF-κB, Sp-1, and AP-1 motifs [2–8]. In the present study, IL-16 up-regulated MMP-9 expression by promoting the activation of NF-κB, Sp-1, and AP-1 binding motifs in VSMCs. In addition, our data indicated that IL-16 induces migration and invasion by VSMCs. Thus, we suggest the novel notion that cytokine IL-16 might mediate MMP-9 expression via the positive regulation of transcription factors NF-κB, Sp-1, and AP-1 binding motifs, which then leads to migration and invasion by VSMCs.

It is now well recognized that signaling pathways regulate the migration and invasion by VSMCs in the presence of growth factors and cytokines [9, 10]. Several lines of studies have demonstrated how IL-16 binds to CD4, and then transduces signaling via the p56^lck^/PI3K/ERK/p38 cascade, protein kinase C phosphorylation, and the STAT6 pathway in several immune cells [22–24]. A number of studies have demonstrated that signaling pathway is strongly linked with transcription factor-mediated MMP-9 regulation in several cell lines [5, 28, 30]. Previous result suggested the transcription factor NF-κB as a main factor in ERK1/2-mediated expression of MMP-9 in TNF-α-treated VSMCs [5, 30]. Other study has shown that p38MAPK signaling is involved in the IL-1β-induced MMP-9 expression via activation of AP-1 in A549 cells [5, 30]. In the present study, our data indicated that phosphorylation of p38MAPK was significantly induced by IL-16 treatment in VSMCs. In addition, p38MAPK mediated MMP-9 expression via the activation of Sp-1 binding, which subsequently led to migration and invasion by VSMCs in response to the binding of IL-16 to CD4. The identification of the p38MAPK pathway as a migratory and invasive signaling molecule that immediately connects cytokine IL-16 and transcription factor Sp-1-mediated MMP-9 expression leading to vascular remodeling may suggest an important potential therapeutic target molecule.

The proliferation of VSMCs is generally regulated by cell-cycle progression after vascular injury [14–18]. However, the role of cell-cycle regulation in the migration and invasion by VSMCs has not been reported. We reveal here the regulatory mechanism of the cell-cycle regulation in the IL-16-induced migration and invasion of VSMCs. In the present study, our data showed that IL-16 did not affect the cell-cycle progression that is responsible for the G1-, S-, and G2/M-phase population in VSMCs. In a thymidine uptake experiment, IL-16 did not induce a proliferation of VSMCs. We next sought to determine whether IL-16 could modulate the expression levels of cell-cycle regulators and tumor-suppressor gene p53 in VSMCs. Results showed that the p21WAF1 expression was significantly up-regulated in IL-16-treated VSMCs, without altering the expression levels of either p27KIP1 or p53. Our data also revealed that the knockdown of p21WAF1 expression abolished the majority of the migration and invasion by VSMCs induced by IL-16, indicating the novel notion that p21WAF1 is a positive key regulator in IL-16-induced migration and invasion by VSMCs. These observations were further supported when results showed that p21WAF1 regulated p38MAPK-mediated MMP-9 expression via the up-regulated binding activation of the Sp-1 motif in the migration and invasion of VSMCs in response to IL-16. These results suggest that p21WAF1 might play a pivotal role in mediating the migration and invasion by IL-16-stimulated VSMCs, which guides to the vascular plaque instability.

In summary, our results provide the first evidence that the binding of IL-16 to CD4 is an essential molecular event that is required for migration and invasion of VSMCs. Our study indicates that p21WAF1 might regulate the p38MAPK signaling pathway via MMP-9 expression by inducing the binding activity of the Sp-1 motif in controlling the migration and invasion of IL-16-stimulated VSMCs, which leads to the formation of vascular lesions in vascular diseases such as atherosclerosis and restenosis. Thus, IL-16 might be act as a novel potential target for the treatment of vascular diseases. Further study is required to examine the efficacy of IL-16 using animal models.

## Supporting Information

S1 Fig Effect of CD4 siRNA (si-CD4-2) on IL-16-induced VSMCs responses. VSMCs were transfected with either si-CD4-2 or scramble siRNA. (A, B) Cells were stimulated with IL-16 for 24 h, and the wound-healing assay and invasion assay was performed. Scale bars represent 400 μm (wound-healing) and 100 μm (invasion). *P < 0.01 compared with IL-16 treatment. (C) Zymography and immunoblot for MMP-9 expression in indicated cells using cell supernatants and cell lysates. For the p38MAPK phosphorylation, transfected cells were stimulated for 10 min, and then cell lysates were subjected to immunoblot. *P < 0.01 compared with IL-16 treatment. (D) EMSA for the binding activity of the Sp-1 motif in either si-CD4-2 or scramble siRNA transfected cells after treatment with IL-16 for 24 h. *P < 0.01 compared with IL-16 treatment. (E) Knockdown efficiency of CD4 siRNA (si-CD4-2) was confirmed by immunoblot in VSMCs. The protein levels were normalized GAPDH. *P < 0.01 compared with control. Results are reported as the means ± SE from three triplicate experiments.
(TIF)

S2 Fig Role of p21WAF1 siRNA (si-p21-2) on IL-16-induced responses of VSMCs. (A, B) **Confluent cells** were transfected with either si-p21-2 or scrambled siRNA, and then stimulated with IL-5 (50 ng/ml) for 24 h, followed by analysis of the wound-healing assay and invasion assay. Scale bars represent 400 μm (wound-healing) and 100 μm (invasion). *P < 0.01 compared with IL-16 treatment. (C) After transfection with either si-p21-2 or scrambled siRNA, the cells were incubated with IL-16 for 24 h, and then cell supernatants and cell lysate were subjected to zymography and immunoblot for the detection of MMP-9 expression. For the p38MAPK signaling, si-p21-2 or scrambled siRNA transfected cells were pretreated with IL-16 for 10 min. The level of p38MAPK phosphorylation was determined in the cell lysates using immunoblot. *P < 0.01 compared with IL-16 treatment. (D) Transfected cells were stimulated with IL-16 for 24 h, and EMSA was performed for the detection of Sp-1 DNA binding activity. (E) The effectiveness of p21WAF1 gene silencing was confirmed using immunoblot. GAPDH was included as a loading control. *P<0.01compared with control. Results are reported as the means±SE from three triplicate experiments.
(TIF)

## References

[1] Ross R. The pathogenesis of atherosclerosis: a perspective for the 1990s. Nature 1993;362: 801–809. 8479518 10.1038/362801a0

[2] Newby AC, Zaltsman AB. Molecular mechanisms in intimal hyperplasia. J Pathol. 2000;190: 300–309. 10685064 10.1002/(SICI)1096-9896(200002)190:3<300::AID-PATH596>3.0.CO;2-I

[3] Cho A, Reidy MA. Matrix metalloproteinase-9 is necessary for the regulation of smooth muscle cell replication and migration after arterial injury. Circ Res. 2002;91: 845–851. 12411400 10.1161/01.res.0000040420.17366.2e

[4] Galis ZS, Johnson C, Godin D, Magid R, Shipley JM, Senior RM, et al Targeted disruption of the matrix metalloproteinase-9 gene impairs smooth muscle cell migration and geometrical arterial remodeling. Circ Res. 2002;91: 852–859. 12411401 10.1161/01.res.0000041036.86977.14

[5] Moon SK, Cha BY, Kim CH. ERK1/2 mediates TNF-alpha-induced matrix metalloproteinase-9 expression in human vascular smooth muscle cells via the regulation of NF-kappaB and AP-1: Involvement of the ras dependent pathway. J Cell Physiol. 2004;198: 417–427. 14755547 10.1002/jcp.10435

[6] Bond M, Rosalind P, Fabunmi P, Baker AH, Newby AC. Synergistic upregulation of metalloproteinase-9 by growth factors and inflammatory cytokines: an absolute requirement for transcription factor NF-kappa B. FEBS Lett. 1998;435: 29–34. 9755853 10.1016/s0014-5793(98)01034-5

[7] Sato H, Seiki M. Regulatory mechanism of 92 kDa type IV collagenase gene expression which is associated with invasiveness of tumor cells. Oncogene 1993;8: 395–405. 8426746

[8] Bond M, Chase AJ, Baker AH, Newby AC. Inhibition of transcription factor NF-kappaB reduces matrix metalloproteinase-1, -3 and -9 production by vascular smooth muscle cells. Cardiovasc Res. 2001;50(3): 556–565. 11376631 10.1016/s0008-6363(01)00220-6

[9] Zhan Y, Kim S, Izumi Y, Izumiya Y, Nakao T, Miyazaki H, et al Role of JNK, p38, and ERK in platelet-derived growth factor-induced vascular proliferation, migration, and gene expression. Arterioscler Thromb Vasc Biol. 2003;23: 795–801. 12637337 10.1161/01.ATV.0000066132.32063.F2

[10] Heldin CH, Westermark B. Mechanism of action and in vivo role of platelet-derived growth factor. Physiol Rev. 1999;79: 1283–1316. 10508235 10.1152/physrev.1999.79.4.1283

[11] Lee SJ, Park SS, Lee US, Kim WJ, Moon SK. Signaling pathway for TNF-alpha-induced MMP-9 expression: mediation through p38 MAP kinase, and inhibition by anti-cancer molecule magnolol in human urinary bladder cancer 5637 cells. Int Immunopharmacol. 2008;8(13–14): 1821–1826. 10.1016/j.intimp.2008.08.018 18801463

[12] Cho A, Graves J, Reidy MA. Mitogen-activated protein kinases mediate matrix metalloproteinase-9 expression in vascular smooth muscle cells. Arterioscler Thromb Vasc Biol. 2000;20: 2527–2532. 11116048 10.1161/01.atv.20.12.2527

[13] Gurjar MV, Deleon J, Sharma RV, Bhalla RC. Role of reactive oxygen species in IL-1 beta-stimulated sustained ERK activation and MMP-9 induction. Am J Physiol Heart Circ Physiol. 2001;281(6): H2568–2574. 11709424 10.1152/ajpheart.2001.281.6.H2568

[14] Chang MW, Barr E, Lu MM, Barton K, Leiden JM. Adenovirus-mediated over-expression of the cyclin/cyclin-dependent kinase inhibitor, p21 inhibits vascular smooth muscle cell proliferation and neointima formation in the rat carotid artery model of balloon angioplasty. J Clin Invest. 1995;96(5): 2260–2268. 7593612 10.1172/JCI118281 PMC185876

[15] Yang ZY, Simari RD, Perkins ND, San H, Gordon D, Nabel GJ, et al Role of the p21 cyclin-dependent kinase inhibitor in limiting intimal cell proliferation in response to arterial injury. Proc Natl Acad Sci USA. 1996;93: 7905–7910. 8755575 10.1073/pnas.93.15.7905 PMC38847

[16] Nathe TJ, Deou J, Walsh B, Bourns B, Clowes AW, Daum G. Interleukin-1beta inhibits expression of p21(WAF1/CIP1) and p27(KIP1) and enhances proliferation in response to platelet-derived growth factor-BB in smooth muscle cells. Arterioscler Thromb Vasc Biol. 2002;22(8): 1293–1298. 12171790 10.1161/01.atv.0000023428.69244.49

[17] Weiss RH, Joo A, Randour C. p21Waf1/Cip1 is an assembly factor required for platelet-derived growth factor-induced vascular smooth muscle cell proliferation. J Biol Chem. 2000;275: 10285–10290. 10744715 10.1074/jbc.275.14.10285

[18] Moon SK, Kim HM, Lee YC, Kim CH. Disialoganglioside (GD3) synthase gene expression suppresses vascular smooth muscle cell responses via the inhibition of ERK1/2 phosphorylation, cell cycle progression, and matrix metalloproteinase-9 expression. J Biol Chem. 2004;279(32): 33063–33070. 15175338 10.1074/jbc.M313462200

[19] Center DM, Cruikshank W. Modulation of lymphocyte migration by human lymphokines. I. Identification and characterization of chemoattractant activity for lymphocytes from mitogen-stimulated mononuclear cells. J Immunol. 1982;128: 2563–2568. 7042840

[20] Cruikshank WW, Berman JS, Theodore AC, Bernardo J, Center DM. Lymphokine activation of T4+ T lymphocytes and monocytes. J Immunol. 1987;138: 3817–3823. 3108375

[21] Richmond J, Tuzova M, Cruikshank W, Center D. Regulation of cellular processes by interleukin-16 in homeostasis and cancer. J Cell Physiol. 2014;229(2): 139–147. 10.1002/jcp.24441 23893766

[22] Ryan TC, Cruikshank WW, Kornfeld H, Collins TL, Center DM. The CD4-associated tyrosine kinase p56lck is required for lymphocyte chemoattractant factor-induced T lymphocyte migration. J Biol Chem. 1995;270: 17081–17086. 7615501 10.1074/jbc.270.29.17081

[23] Parada NA, Cruikshank WW, Danis HL, Ryan TC, Center DM. IL-16- and other CD4 ligand-induced migration is dependent upon protein kinase C. Cell Immunol. 1996;168: 100–106. 8599832 10.1006/cimm.1996.0054

[24] Liu C, Mills J, Dixon K, Vennarini J, Cunningham M, Del Vecchio A, et al IL-16 signaling specifically induces STAT6 activation through CD4. Cytokine 2007;38: 145–150. 17624801 10.1016/j.cyto.2007.05.016

[25] Cross DS, McCarty CA, Hytopoulos E, Beggs M, Nolan N, Harrington DS, et al Coronary risk assessment among intermediate risk patients using a clinical and biomarker based algorithm developed and validated in two population cohorts. Curr Med Res Opin. 2012;28(11): 1819–1830. 10.1185/03007995.2012.742878 23092312 PMC3666558

[26] Yoon TJ, Yu KN, Kim E, Kim JS, Kim BG, Yun SH, et al Specific Targeting, Cell Sorting, and Bioimaging with Smart Magnetic Silica Core–Shell Nanomaterials. Small 2006;2(2): 209–215. 17193022 10.1002/smll.200500360

[27] So MK, Loening AM, Gambhir SS, Rao J. Creating self-illuminating quantum dot conjugates. Nat Protoc. 2006;1(3): 1160–1164. 17406398 10.1038/nprot.2006.162

[28] Lee SJ, Cho SC, Lee EJ, Kim S, Lee SB, Lim JH, et al Interleukin-20 promotes migration of bladder cancer cells through extracellular signal-regulated kinase (ERK)-mediated MMP-9 protein expression leading to nuclear factor (NF-κB) activation by inducing the up-regulation of p21(WAF1) protein expression. J Biol Chem. 2013;288(8): 5539–5552. 10.1074/jbc.M112.410233 23271730 PMC3581371

[29] Bendeck MP, Zempo N, Clowes AW, Galardy RE, Reidy MA. Smooth muscle cell migration and matrix metalloproteinase expression after arterial injury in the rat. Circ Res. 1994;75: 539–545. 8062427 10.1161/01.res.75.3.539

[30] Lin CC, Kuo CT, Cheng CY, Wu CY, Lee CW, Hsieh HL, et al IL-1 beta promotes A549 cell migration via MAPKs/AP-1- and NF-kappaB-dependent matrix metalloproteinase-9 expression. Cell Signal. 2009;21(11): 1652–62. 10.1016/j.cellsig.2009.07.002 19616091

