# Supplementary figures and images for: p21WAF1 Is Required for Interleukin-16-Induced Migration and Invasion of Vascular Smooth Muscle Cells via the p38MAPK/Sp-1/MMP-9 Pathway
Source: PLoS One. 2015 Nov 6;10(11):e0142153. doi: 10.1371/journal.pone.0142153 (PMC4636239; doi:10.1371/journal.pone.0142153)

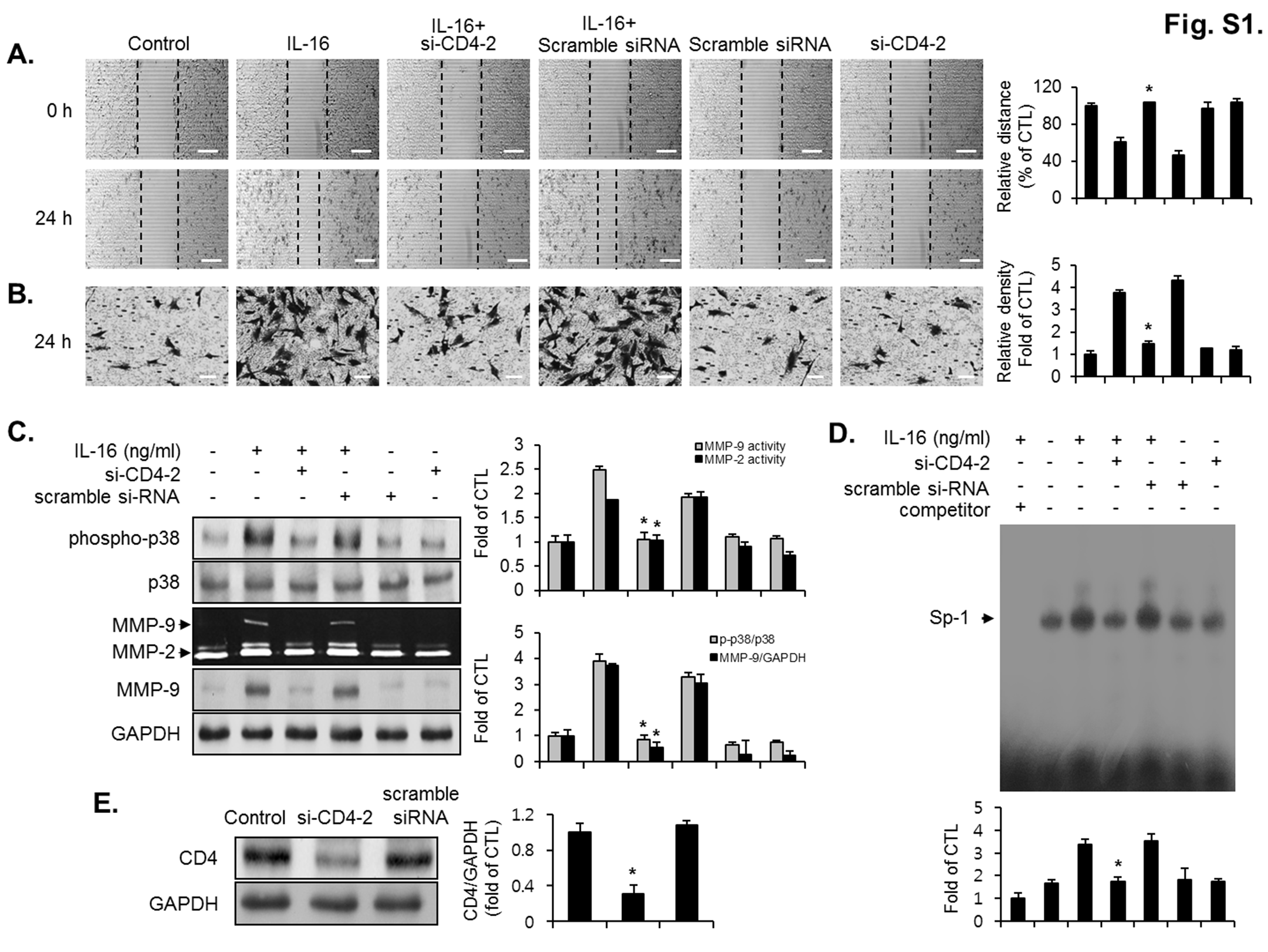

Supplement: S1 Fig — VSMCs were transfected with either si-CD4-2 or scramble siRNA. (A, B) Cells were stimulated with IL-16 for 24 h, and the wound-healing assay and invasion assay was performed. Scale bars represent 400 μm (wound-healing) and 100 μm (invasion). *P < 0.01 compared with IL-16 treatment. (C) Zymography and immunoblot for MMP-9 expression in indicated cells using cell supernatants and cell lysates. For the p38MAPK phosphorylation, transfected cells were stimulated for 10 min, and then cell lysates were subjected to immunoblot. *P < 0.01 compared with IL-16 treatment. (D) EMSA for the binding activity of the Sp-1 motif in either si-CD4-2 or scramble siRNA transfected cells after treatment with IL-16 for 24 h. *P < 0.01 compared with IL-16 treatment. (E) Knockdown efficiency of CD4 siRNA (si-CD4-2) was confirmed by immunoblot in VSMCs. The protein levels were normalized GAPDH. *P < 0.01 compared with control. Results are reported as the means ± SE from three triplicate experiments. (TIF) [file pone.0142153.s001.tif]

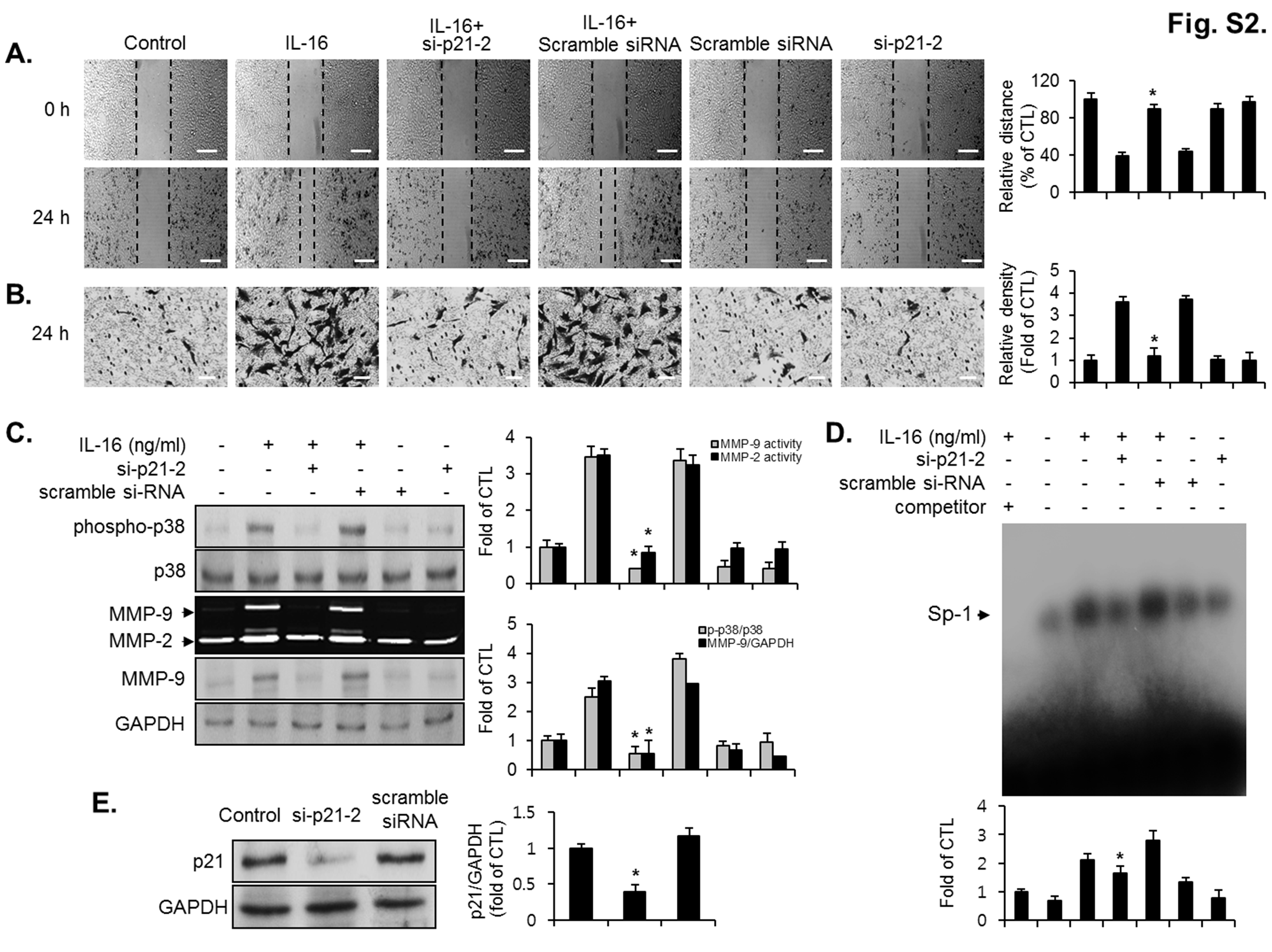

Supplement: S2 Fig — (A, B) Confluent cells were transfected with either si-p21-2 or scrambled siRNA, and then stimulated with IL-5 (50 ng/ml) for 24 h, followed by analysis of the wound-healing assay and invasion assay. Scale bars represent 400 μm (wound-healing) and 100 μm (invasion). *P < 0.01 compared with IL-16 treatment. (C) After transfection with either si-p21-2 or scrambled siRNA, the cells were incubated with IL-16 for 24 h, and then cell supernatants and cell lysate were subjected to zymography and immunoblot for the detection of MMP-9 expression. For the p38MAPK signaling, si-p21-2 or scrambled siRNA transfected cells were pretreated with IL-16 for 10 min. The level of p38MAPK phosphorylation was determined in the cell lysates using immunoblot. *P < 0.01 compared with IL-16 treatment. (D) Transfected cells were stimulated with IL-16 for 24 h, and EMSA was performed for the detection of Sp-1 DNA binding activity. (E) The effectiveness of p21WAF1 gene silencing was confirmed using immunoblot. GAPDH was included as a loading control. *P<0.01compared with control. Results are reported as the means±SE from three triplicate experiments. (TIF) [file pone.0142153.s002.tif]
